# Undifferentiated high-grade pleomorphic sarcoma of ethmoid sinus: a case report and literature review^☆^

**DOI:** 10.1016/j.bjorl.2017.05.004

**Published:** 2017-06-01

**Authors:** Yupeng Zhu, Dapeng Hao, Xiaoyan Tang, Lei Sun

**Affiliations:** Affiliated Hospital of Qingdao University, Department of Radiology, Qingdao, China

## Introduction

Undifferentiated high-grade pleomorphic sarcoma (UHPS) is also known as malignant fibrous histiocytoma (MFH). It is recognized as one of the most common soft tissue sarcomas arising in late adulthood,^1^ accounting for 20–30% of all soft-tissue sarcomas.^2^ Most UHPSs arise from the extremities and the retroperitoneum, and the incidence of head and neck UHPS is relatively low. Only 3–10% of all UHPSs occur in the head and neck region.^2^, ^3^ Sinonasal UHPSs are even rarer. Most sinonasal UHPSs are found in maxillary sinus and two cases respectively ethmoid sinus and frontal sinus are reported.

## Case report

The patient was a 61-year-old woman with a neoplasm in the right nasal cavity for two months with in addition a 20 year history of nasal polyp in the right nasal cavity. She also presented with a two-month history of headache on the right side with tightness, tears in the right eye and blurred vision, as well as hyposmia and hearing loss. Episodes of epistaxis gradually increased in both, the frequency and amount of bleeding. The ophthalmic testing is not unusual.

**Figure 1:**
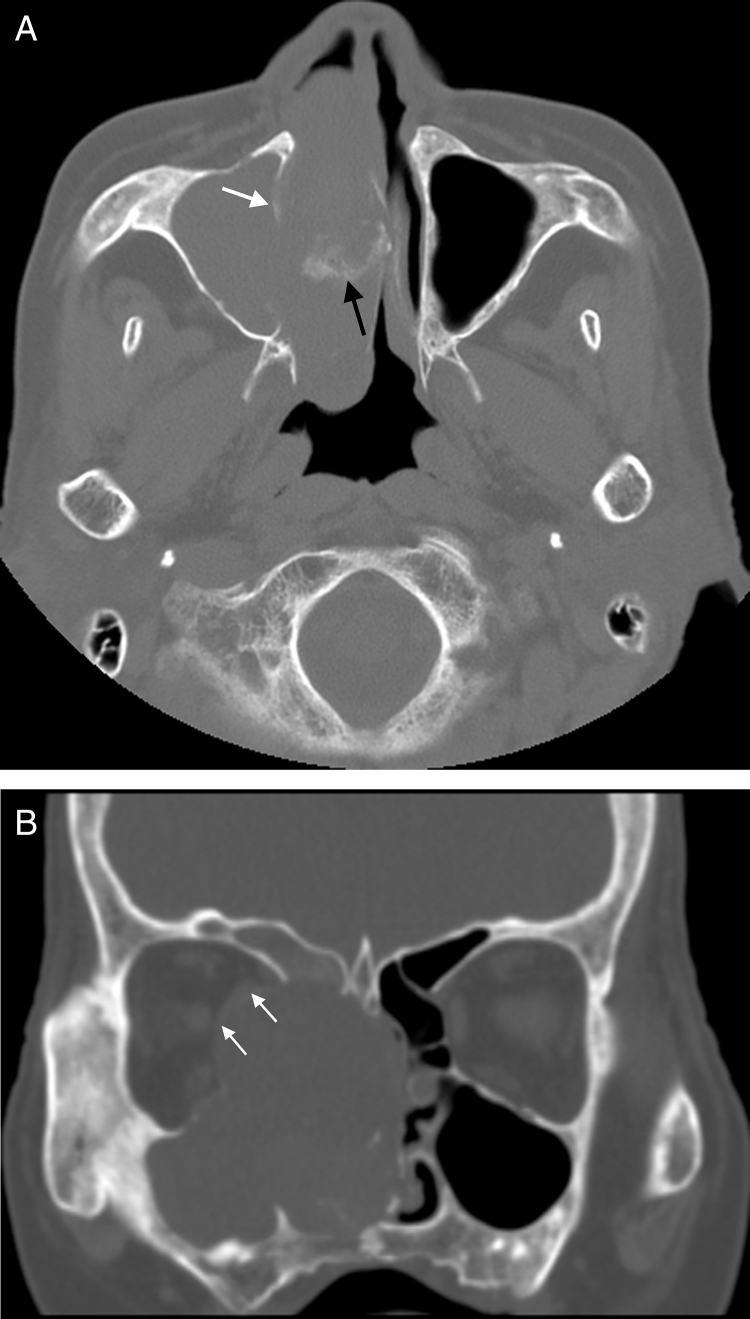
(A) Axial CT scan shows the soft tissue mass in the paranasal sinus and nasal cavity. Bone destruction of the medial wall of maxillary sinus is apparent and the margin is obscure (white arrow in A). Patchy calcification is found in the lesion (black arrow in A). (B) Coronal CT scan shows main body of soft-tissue mass is located in ethmoid sinus and nasal cavity and it damaged bone of right orbit with compression of the medial rectus (white arrow in B). The wall of the right ethmoidal sinus, right maxillary sinus and right frontal sinus were damaged.

**Figure 2:**
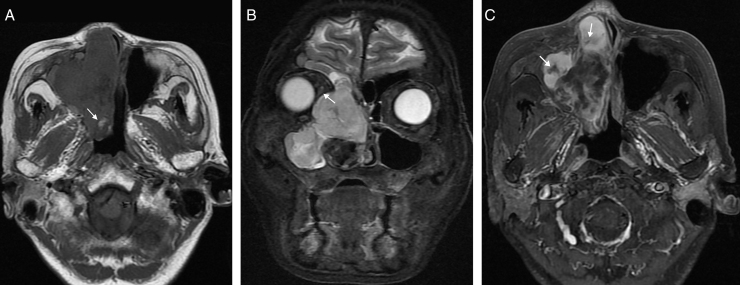
(A) Axial T1-weighted image shows lower-signal intensity and high-signal intensity. The surrounding structures are compressed. Hemorrhagic focus can be found in the mass (white arrow in A). (B) Coronal T2WI with fat suppression shows the lesion more obviously. The lesion involved the right ethmoidal sinus, right maxillary sinus, right frontal sinus and right nasal cavity. Medial rectus was compressed to the right side by the lesion (white arrow in B). (C) Contrast-enhanced T2-weighted image with fat-suppression shows heterogeneous enhancement of the soft tissue mass and striped dis-enhanced region (white arrow in C).

Diagnostic imaging included CT and MRI. The coronal and axial CT images showed a soft-tissue mass of right ethmoidal sinus, involving the right maxillary sinus, right frontal sinus and right nasal cavity. Bone erosion was observed in the right lamina of ethmoidal cells. The soft-tissue mass invaded the bony wall of right orbit and compressed the right eyeball (Fig. 1). MRI demonstrated the same appearances (Fig. 2). In additional, the mucous membrane of left ethmoidal sinus and bilateral sphenoid sinus were also compromised. Contrast-enhanced MRI confirmed heterogeneous enhancement of the lesion. Striped low-signal intensity was seen in the lesion.

With the aggravating clinical symptoms, age and image features of CT and MRI, clinicians and radiologists diagnosed this soft-tissue mass as sinonasal malignant tumor. According to the diagnosis of CT and MRI, the malignant tumor originated from ethmoid sinus involved frontal sinus, maxillary sinus, sphenoid sinus and orbit without neck lymph nodes or distant metastases. Based on the above a few, the TNM staging was T_4_N_0_M_0_.

With a primary diagnosis of sinonasal malignant tumor, the otolaryngologists performed a tumor resection for the patient. The operation involved tumor resection of nasal cavity, paranasal sinus and orbit and endoscopic full sinus surgery. Under general anesthesia, the whole soft tissue mass in the paranasal sinus and nasal cavity was resected. In addition they resected residuary inferior nasal concha, concha nasalis media and superior nasal concha.

**Figure 3:**
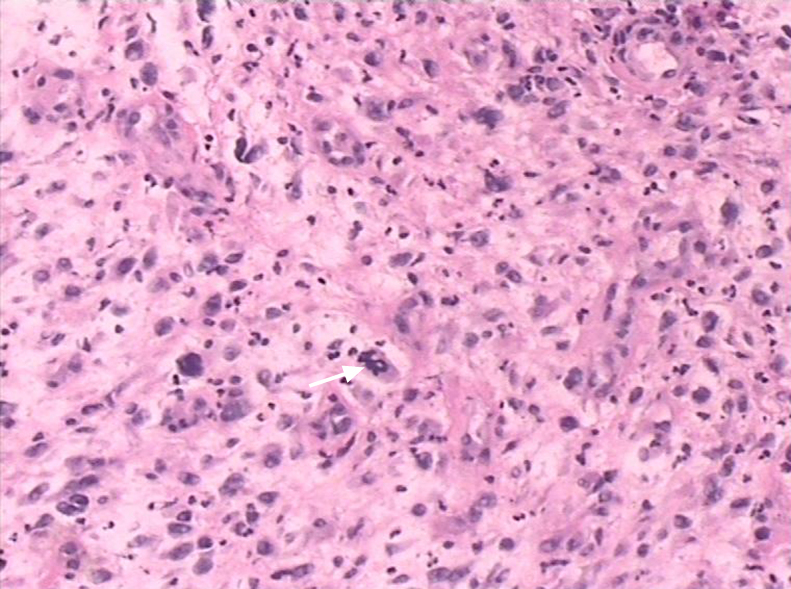
Pathological analysis of UHPS showed that spindle cell bestrewed in the horizon (hematoxylin–eosin staining, original magnification: 10× objective lenses). Multinuclear giant cell can be founded in the background of spindle cell (white arrow).

Fast frozen pathology examination during the operation showed the neoplasm was borderline or low-grade malignant tumor. Histologic examination showed that spindle cell bestrewed in the horizon (Fig. 3). The stroma was loose. Immunohistochemistry staining was positive for Vimentin and it was negative for CD68, CD34, CD99, Bcl-2, Actin, S-100, SMA and Desmin. The tumor was rich in dysplastic cells. The multinuclear giant cell could be founded in the background of spindle cell. With these immunohistochemical indexes and image feature, pathologists diagnosed the tumor as UHPS.

Neither adjuvant chemotherapy or radiation therapy was used in the therapeutic process. Clinicians gave anti-infective therapy for this patient with Ceftriaxone after surgery. With 16 and 28 months of surveillance after leaving the hospital, the patient remained asymptomatic. Enhanced MR scanning showed no sign of tumor recurrence.

## Discussion

High-grade pleomorphic malignant tumors that lack a specific line of differentiation are classified as “undifferentiated high-grade pleomorphic sarcoma/malignant fibrous histiocytoma”.^4^

Squamous cell carcinoma and lymphoma are the most common malignancies in the head and neck (80–90%). Sarcomas are relatively rare, accounting for 1–11% of all neoplasms in this area.^5^ In the head and neck, UHPS occurs most commonly in the maxillary sinus, followed by the ethmoid sinus, nasal cavity, sphenoid sinus, and frontal sinus.^1^, ^6^ Most patients are between 50 and 70 years of age, and men are affected 2–3 times as commonly as women.^1^ In our case, the patient is a 61-year-old female.

Clinically, patients with UHPS usually present with an enlarging, painless, solid soft-tissue mass.^1^ The most common presentation for UHPS occurring in paranasal sinus includes nasal airway obstruction, pain, epistaxis and hypoesthesias. If optic nerve is compressed, patients will have several symptoms of blurred vision and double image. UHPS may encroach upon the orbit resulting in exophthalmos.

Most of the tumors showed nonspecific various signal intensity on CT and MR images.^1^, ^7^ CT can help evaluate bone destruction and sinonasal soft masses. MRI is the best tool to delineate soft tissue extension and compression of surrounding tissues.

The most common CT features are aggressive bone destruction and compressive bony absorption of sinus wall. The lesion easily erodes ipsilateral orbital medial wall in ethmoidal cells. In our case, ipsilateral orbital medial was destroyed and medial rectus and optic nerve were compressed to right side. On CT, the soft tissue mass shows isodensity or low density. Calcification foci can be found in some lesions.^1^ Striped calcification was found in our case. The tumor shows inhomogeneous iso-hyperintensity on T1WI, and mixed signal on T2WI. The collagenous fiber is hypointense while the liquefaction necrosis and mucoid degeneration are hyperintense on T2WI. Contrast-enhanced MRI confirmed homogeneous enhancement of the solid portion of lesion. The liquefaction necrosis and cystic degeneration are more significant in UHPS than other soft tissue tumors. The bigger the mass is, more common liquefaction necrosis and cystic degeneration are.^8^

Based on our case and literatures,^1^, ^2^, ^3^, ^5^, ^6^ we found the following imaging features of UHPS in the paranasal sinuses and nasal cavity: (i) the lesion fills up the whole paranasal sinus, (ii) aggressive bone destruction of sinus wall, (iii) erodes ipsilateral orbital medial wall, (iv) collagenous fiber, (v) homogeneous enhancement of the solid portion of lesion, (vi) liquefaction necrosis and cystic degeneration, and (vii) calcification.

Definitive diagnosis of UHPS relies on pathological assessment.^5^ In addition to light microscopy,electron microscopy and immunohistochemical can be used.

The differential diagnosis for sinonasal UHPS includes squamous carcinoma, hemorrhagic and necrotic polyps, and inverted papilloma. CT shows obviously uneven density and extensive bone destruction in the squamous carcinoma. Hemorrhagic and necrotic polyps involve nasal cavity and maxillary sinus at the center of maxillary sinus. Contrast-enhanced imaging of it shows flocculent and patchy enhancement in the mass. Bony sclerosis is obvious in the wall of the maxillary sinus. Inverted papilloma is usually lobular. Contrast-enhanced MRI shows gyriform enhancement.^9^

The prognosis of UHPS arising from head and neck has been reported to be generally poorer when compared with UHPS in other regions.^10^ In particular, the prognosis of tumors arising from the sinonasal tract is reported to be worse than those from other parts of the head and neck region. Furthermore, the prognosis of repeat operations for recurrences of head and neck UHPS has been reported to be poor, offering a “cure rate” of 23%.^10^ According to the literature, the overall 5 year survival rate is approximately 50–60% for the UHPS of the head and neck.

## Conclusion

UHPS of paranasal sinus and orbit is very rare. CT and MRI were useful for tumor delineation. Imaging findings suggested malignancy, but are nonspecific to UHPS. On MRI, the tumor shows inhomogeneous signal intensity, which may reflect the complex histologic components of the tumor.

## Conflicts of interest

The authors declare no conflicts of interest.
